# MyoD1 suppresses cell migration and invasion by inhibiting FUT4 transcription in human gastric cancer cells

**DOI:** 10.1038/s41417-019-0153-3

**Published:** 2019-12-12

**Authors:** Fei Wu, Yannan Qin, Qiuyu Jiang, Jinyuan Zhang, Fang Li, Qian Li, Xiaofei Wang, Yi Gao, Jiyu Miao, Chen Guo, Yang Yang, Lei Ni, Liying Liu, Shuqun Zhang, Chen Huang

**Affiliations:** 1 Department of cell Biology and Genetics, School of Basic Medical Sciences, Xi’an Jiaotong University Health Science Center, Xi’an, 710061 China; 2 Department of Oncology, The Second Affiliated Hospital of Xi’an Jiaotong University, Xi’an, 710004 China; 3 Key Laboratory of Environment and Genes Related to Diseases (Xi’an Jiaotong University), Ministry of Education of China, Xi’an, 710061 Shaanxi China; 4 Department of Cell Biology and Genetics, Medical College of Yan’an University, Yan’an, 716000 Shaanxi Province China; 5 School of Public Health, Xi’an Jiaotong University Health Science Center, Xi’an, Shaanxi 710061 China

**Keywords:** Gastric cancer, Gastric cancer

## Abstract

Myogenic differentiation 1 (MyoD1) is a transcription factor that promotes expression of muscle-specific genes. MyoD1 is expressed at significantly lower levels in gastric cancer (GC) tissues and cells, and it induces apoptosis in GC cells. However, functions for MyoD1 in GC cell migration and gene expression have not been documented. We show that knockdown of MyoD1 promoted migration and invasion of GC cells, whereas MyoD1 overexpression suppressed migration and invasion. We performed chromatin immunoprecipitation (ChIP)-sequencing to identify MyoD1 target genes in MKN-45 cells. The 2-kb upstream regions (Up2k) of the transcription start sites of 57 genes were probably bound by MyoD1. Six of these genes function in signaling pathways such as synthesis of glycosphingolipid biosynthesis—lacto and neolacto series. MyoD1 inhibited transcription of fucosyltransferase IV (FUT4) by binding directly to the *FUT4* F3; this finding was validated by ChIP-quantitative PCR and a luciferase reporter assay. Ulex europaeus agglutinin I, which binds Fucα1-2Galβ1-4GlcNAc, and Lewis antigens showed decreased binding to the plasma membrane of cells that overexpressed MyoD1. Knockdown of FUT4 mimicked MyoD1 overexpression by suppressing GC cell migration and invasion; this result implied that MyoD1 suppressed cell migration and invasion via inhibiting the FUT4/matrix metallopeptidase signaling pathway. In summary, this study demonstrated that MyoD1 suppresses migration and invasion of GC cells by directly binding to the F3 region in the *FUT4* Up2k and inhibiting FUT4/type II Lewis antigen expression.

## Introduction

The myogenic differentiation (MyoD) family of transcription factors is composed of a group of related basic helix–loop–helix DNA-binding proteins, which includes MyoD1/myf-3, myogenin/myf-4, myf-5, and myf-6 (MRF4, herculin) [1, 2]. MyoD1 is a transcriptional activator that promotes the expression of muscle-specific genes and functions in muscle differentiation together with MYF5 and MYOG [3]. MyoD1 can also work with a transient placeholder protein that functions to prevent other transcription factors from binding to the DNA and also retains an inactive conformation for the DNA [4]. One of the main actions of MyoD is enhancing transcription of p21 and myogenin to remove cells from the cell cycle and halt proliferation of differentiated myocytes [5]. In general, high MyoD1 expression represses cell renewal, promotes terminal differentiation, and can induce apoptosis in myoblasts.

Expression and function of MyoD1 are dysregulated in medulloblastoma [6], retinoblastoma [7], lung adenocarcinoma [8], head and neck cancer [9], and breast cancer [10]. For example, MyoD1 functions cooperatively with the retinoblastoma tumor suppressor gene to cause cell cycle arrest in the terminally differentiated myoblasts [7]. We have shown that MyoD1 expression was significantly lower in gastric cancer (GC), and gradually decreased in G1, G2, and G3 GC tissues. The promoter region of *MyoD1* contains CpG islands that were hypermethylated and bound with MeCP2, which suppressed GC cell apoptosis by inhibiting the MYOD1/caspase-3 signaling pathway [11]. However, little is known about the precise function of MyoD1 in migration and invasion, and interaction with genes in GC cells.

Fucosyltransferase IV (FUT4) is the key enzyme for the synthesis of type II Lewis antigen (LeY, LeX, and sLeX) carried by glycoproteins and glycolipids on cell membranes. High expression of FUT4 has been found in different types of cancers, including acute lymphoblastic leukemia, colon, breast, pancreatic, lung, and GCs [12–16]. Down-regulation of FUT4 inhibits epithelial–mesenchymal transition (EMT) and invasion of lung cancer by inactivating epidermal growth factor receptor and blocking mitogen-activated protein kinase and nuclear factor-κB (NF-κB) signaling pathways [13]. FUT4 induced activation of phosphatidylinositol 3-kinase and inactivated glycogen synthase kinase (GSK3β) and nuclear translocation of NF-κB, resulting in increased Snail and matrix metallopeptidase-9 (MMP-9) expression and greater cell motility. Thus, FUT4 is a novel regulator of EMT in breast cancer cells [12]. In GC, FUT4 was highly expressed on gastric cell surfaces, and this expression was regulated by transcription factors HSF1 and SP1 [17].

In this study, we examined the in vitro migration and invasion abilities of GC cell lines after transfecting small interfering RNA (siRNA) or a MyoD1 overexpression plasmid. In addition, we constructed lentiviral vectors containing full-length human *MYOD1* DNA (Hanbio. Co. Ltd) to overexpress MyoD1 in MKN-45 cells and performed tumor metastasis assays with the cells in mice. The MyoD1 target genes were identified and validated by chromatin immunoprecipitation-sequencing (ChIP-Seq) and luciferase reporter assays. Transcription of *FUT4* and expression of Ulex europaeus agglutinin I (UEA-I) binding glycopattern were inhibited by MyoD1, which bound to the promoter region of *FUT4*, and GC cell migration and invasion were suppressed via FUT4/MMP signaling pathway.

## Materials and methods

### Cell culture

Human GC cell lines BGC-823, MKN-45, and SGC-7901 were obtained from the Cell Bank (Shanghai Genechem Co., Ltd., Shanghai, China). All cell lines were authenticated by the Cell Bank. For verification, mycoplasma tests were performed in our laboratory, and cell morphology and behavior were consistent with the Cell Bank descriptions. Cells (1 × 10^5^ cells/ml) were cultured in RPMI-1640 medium (Gibco-BRL, NY, USA) supplemented with 10% fetal bovine serum (Gibco) at 37 °C in a humidified incubator containing 5% CO_2_.

### siRNA synthesis, plasmid construction, and transfection

siRNAs were pre-designed for *MYOD1* and *FUT4* gene silencing; the siRNAs were synthesized by GenePharma Corporation (SGC, Shanghai, China). A scrambled sequence siRNA was used as a negative control (NC-siRNA). The siRNA sequences are listed in Table 1. Full-length human *MYOD1* complementary DNA was cloned into pCMV2-GV146 vector (Genechem Co. Ltd., Shanghai, China). After culturing SGC-7901 cells for 24 h in plates, the siRNAs were transfected into the cells using Lipofectamine TM-2000 (Invitrogen) according to the manufacturer’s protocol. BGC-823 and MKN-45 cells were seeded in RPMI-1640 medium without antibiotics for 24 h. Then, the pCMV2-GV146 vector or pCMV2-GV146-*MYOD1* vector was transiently transfected into the cells using Lipofectamine 2000 (Invitrogen, Carlsbad, CA, USA). Transfected cells were cultured for 24–48 h before performing assays.

**Table 1:** All primers sequences and oligonucleotide sequences.

Primer name	Sequence	
Primers for qRT-PCR		
β-Actin F	CCAACCGCGAGAAGATGA	
β-Actin R	CCAGAGGCGTACAGGGATAG	
MyoD1-F	AGCACTACAGCGGCGACT	
MyoD1-R	GCGACTCAGAAGGCACGTC	
FUT4-F	AAGGTCCAGGCCCACTGAAG	
FUT4-R	CAGTTCAGGTGACAGAGGCTCAA	
Oligonucleotide sequences for siRNA		
Negative siRNA (NC-siRNA) sense	UUCUCCGAACGUGUCACGUTT	
Negative siRNA (NC-siRNA) antisense	ACGUGACACGUUCGGAGAATT	
MYOD1 siRNA sense	GUAAAUGAGGCCUUUGAGATT	
MYOD1 siRNA antisense	UCUCAAAGGCCUCAUUUACTT	
FUT4 siRNA-1 sense	CAUGUGACCGUGGACGUGUTT	
FUT4 siRNA-1 antisense	ACACGUCCACGGUCACAUGTT	
FUT4 siRNA-2 sense	CUCGCAGCACCUGGAUUAUTT	
FUT4 siRNA-2 antisense	AUAAUCCAGGUGCUGCGCGAGTT	
Primers for ChIP		
Primer 1		
Primer 1-F	CTGTCTTTACACCCTTAACTTGG	
Primer 1-R	TATTGCCTGTTCAGTGAGATG	
Primer 2		
Primer 2-F	TTATGAGTCCTGTGCGTCTT	
Primer 2-R	CCAGCCCAGTAAAGGATAGA	
Primer 3		
Primer 3-F	GGGCTGGCAGGCAAAGGAAA	
Primer 3-R	CCCGCCGAGACGGTCGAATT	

### RNA extraction and qRT-PCR

Total RNA from the cell lines was extracted using TRIzol reagent (Invitrogen, Carlsbad, CA, USA) according to the manufacturer’s instructions. RNA concentrations were measured spectrophotometrically using Nanodrop (Thermo Fisher Scientific Inc., DE, USA). Complementary DNA was synthesized according to the manufacturer’s protocol (Takara, Dalian, China). Quantitative real-time polymerase chain reaction (qRT-PCR) was performed using the SYBR Green PCR Kit (Takara Biotechnology, Takara, Dalian, China). The primers are listed in Table 1. All qRT-PCR reactions were performed in triplicate for each sample using an IQ5 Multicolor qRT-PCR Detection System (Bio-Rad, USA). The control messenger RNA (mRNA) was β-actin. The 2−ΔΔCt method was employed in the qRT-PCR analysis.

### Western blotting

GC cell protein lysates were separated in 10% sodium dodecyl sulfate-polyacrylamide gel electrophoresis, transferred to polyvinylidene difluoride membranes (Roche, Indianapolis, IN, USA), and then probed with antibodies and a commercial ECL Kit (Pierce, Rockford, IL, USA). Protein loading was estimated by using human anti-β-actin monoclonal antibody (Bioworld Biotechnology). Luminescent signals were recorded by Syngene GBox (Syngene, Cambridge, UK). The primary and secondary antibodies used are listed as follows: anti-β-actin monoclonal antibody (Bioworld Biotechnology, BS6007M), MyoD1 (GeneTex, GTX100885), MMP-2 (Proteintech, 10373-2-AP), MMP-9 (Proteintech, 10375-2-AP), vimentin (Cell Signaling Technology, 5741S), N-cadherin (Cell Signaling Technology, 22018-1-AP), Flag (Proteintech, 20543-1-AP), FUT4 (Proteintech, 19497-1-AP), glyceraldehyde 3-phosphate dehydrogenase (Proteintech, 60004-1-Ig), goat anti-mouse immunoglobulin G (IgG) (Proteintech, SA00001-1), and goat anti-rabbit IgG (Proteintech, SA00001-2).

### Wound-healing assay

A wound-healing assay was performed to measure cell migration capacity. Briefly, once cells had grown to 80% confluence in 24-well plates, a single scratch wound was generated with a 10-μl disposable pipette tip. The extent of wound closure was measured at 24, 36, and 48 h after wounding.

### Transwell assays

Transwell chambers (8-μm pore size; Millipore, Billerica, MA, USA) were coated with Matrigel (15 μg/filter; BD Biosciences, Franklin Lakes, NJ, USA). Cells (2.0 × 10^4^) or not in serum-free medium were plated into the upper chamber, and the bottom wells were filled with complete medium. The cells were allowed to invade across the Matrigel-coated membrane for 48 h. Following incubation, the cells were removed from the upper surface of the filter by scraping with a cotton swab. The invaded cells that adhered to the bottom of the membrane were stained with 0.1% crystal violet.

### Lentiviral construction and cell transfection

Lentiviral vectors containing full-length human *MYOD1* DNA (Hanbio. Co. Ltd) were used to overexpress MyoD1 in BGC-823 and MKN-45 cells (LV-MyoD1-BGC-823 and LV-MyoD1-MKN-45). Both cells were seeded in 6-well plates and infected with 1 ml viral stock for 10 h at 37 °C, after which the medium was replaced with normal culture medium. The efficiency of MyoD1 overexpression was determined by qRT-PCR and immunoblotting.

### Tumor metastasis assay in nude mice

Five-week-old male BALB/c nude mice (Vitalriver, Beijing China) were used to analyze tumor metastasis. We randomly numbered six mice, used random number generation to divide mice into two groups (LV-MyoD1-MKN-45 and control (LV-CN)), three mice per group. The number of mice was in accordance with the minimum standards for experimental statistics and along with the Approval for Research Involving Animals. MKN-45 cells were transfected with lentiviral vector LV-MyoD1-MKN-45 and control (LV-CN) and re-suspended in phosphate-buffered saline (PBS); then, 1 × 10^6^ cells were injected into mice by tail vein. Tumors were examined by bioluminescent imaging using IVIS Spectrum (Xenogen Corp., Alameda, CA, USA) 21 days after injection. Briefly, the luciferin substrate was injected into the intraperitoneal cavity at a dose of 150 mg/kg body weight (30 mg/ml luciferin), ~15 min before imaging. Mice were anesthetized with isoflurane/oxygen and placed on the imaging stage. After sacrificing the mice, images of metastasis-bearing lungs and other organs were taken with IVIS Spectrum. All animal experiments were approved by the Institutional Animal Care and Use Committee of Xi’an Jiaotong University, China (No.: 2019-1210).

### ChIP-Seq and ChIP-qRT-PCR

ChIP was conducted as described [18]. Briefly, MKN-45 cells were cross-linked with 1% formaldehyde for 15 min at room temperature and quenching was performed with glycine (125 mmol/L). Nuclear lysates were sonicated using a cell cracker. The chromatin was sonicated into 200-bp (approx.) fragments. The lysates were divided into two portions and incubated, respectively, with 5 μg antibodies against MyoD1 or IgG (Abcam, Cambridge, MA, USA) overnight at 4 °C. DNA–protein complexes were captured using Dynabeads Protein A (Invitrogen, Carlsbad, CA, USA) and eluted in TE buffer at 65 °C. Crosslinking was reversed for 8 h at 65 °C. DNA was extracted using the QIA Quick PCR Purification Kit (Qiagen, Germany) and sequenced on Illumina HiSeq 2000 using the TruSeq Rapid SBS Kit (Illumina, USA, FC-402-4002), according to the manufacturer’s instructions. The locations of ChIP-enriched DNA present in the library were based on the Human Feb 2009 assembly and visualized using the genome browser of the University of California. Peak calling in the mapped ChIP-Seq data was performed with ChIP-Peak and subjected to further bioinformatics analysis. ChIP-Seq experiments were conducted by KangChen Bio-Tech (KangChen, Shanghai, China), and analysis of DNA via qRT-PCR or RT-PCR was performed using gene-specific primers (Table 1).

### Luciferase reporter assay

The promoter region of *FUT4* containing MyoD1-binding sites (MBSs) was cloned downstream of the luciferase reporter in the pGL3-Promoter Vector, between *Kpn*l and *Xho*I sites. All procedures were performed as described [19]. The vector was co-transfected with pCMV2-GV146 vector, or pCMV2-GV146-*MYOD1* vector into HEK293 cells. The reporter gene assays were performed 48 h post transfection using the Luciferase Reporter assay system (Promega), according to the manufacturer’s instructions. Then, the luciferase activity per 1000 cells (Trypan blue staining) was measured. The normalized firefly luciferase activity (firefly luciferase activity/Renilla luciferase activity) for each construct was compared with that of the pGL3-Promoter vector control. The normalized firefly luciferase activity was also compared in pCMV2-GV146 vector or pCMV2-GV146-*MYOD1* vector-transfected cells. All experiments were performed at least three times.

### Lectin microarray and data analysis

A lectin microarray was produced and incubated with Cy3 fluorescent dye-labeled (GE Healthcare) cell total proteins according to our protocol [20–22] with some modifications. Thirty-seven lectins with different binding preferences covering N- and O-linked glycans were spotted onto Jingxin® optical grade epoxysilane-coated slides using PersonalArrayer 16 (CapitalBio, Beijing, China). After immobilization, the slides were blocked with buffer containing 2% bovine serum albumin (BSA) in PBS (0.01 mol/L phosphate buffer containing 0.15 mol/L NaCl, pH 7.4) for 1 h, and then washed with PBST (0.2% Tween-20 in PBS) and PBS twice each for 5 min before drying. Six micrograms of total Cy3 protein [20–22] was diluted in 0.5 mL of incubation buffer containing 2% (w/v) BSA, 500 mmol/L glycine, and 0.1% Tween-20 in PBS. Then, the sample was incubated with the blocked lectin microarray at 25 °C for 3 h in a rotisserie oven set at 4 r.p.m. Slides were washed with PBST and PBS twice each for 5 min and dried by centrifugation at 600 r.p.m. for 5 min. The microarrays were scanned with a 70% photomultiplier tube at a 50% laser power setting with LuxScan 10K-B confocal scanner (CapitalBio, Beijing, China). The acquired images were analyzed at 532 nm. The averaged background was subtracted, and values less than the average background ± 2 standard deviations (SD) were removed from each data point. The median of the effective data point for each lectin was globally normalized to the sum of the median of all effective data points for each lectin in a block. Each sample was observed consistently with three repeated slides, and the normalized median of each lectin from nine repeated blocks was averaged and the SD determined. Normalized data for the control and MyoD1-overexpressed cell were compared according to the following criteria: fold change ≥2.0 or ≤0.5 indicated up-regulation or down-regulation. Differences between the two arbitrary data sets were tested by paired Student’s *t* test using SPSS Statistics 19.

### Lectin immunofluorescence

Lectins labeled by Cy3 fluorescent dye were applied to detect the specific sugar structure in cells according to our protocol [20]. Cells (2 × 10^5^) were seeded in 6-well culture plates containing sterile coverslips. The treated adherent cells were fixed with 4% paraformaldehyde and permeabilized in ice-cold 1× PBS supplemented with 1% Triton X-100 at 4 °C for 10 min and rinsed twice in 1× PBS. Prior to incubation with the labeled lectin, the fixed cells were blocked with the buffer (1× PBS supplemented with 4% BSA) at 37 °C for 30 min. The cells were incubated with the labeled lectin diluted to a final concentration of 15–20 μg/mL with the blocking buffer for 3 h at room temperature in the dark. Then, they were further stained with 1 μg/mL of DAPI (4′,6-diamidino-2-phenylindole) (Roche, Basel, CH) in 1× PBS for 10 min and a final rinse was performed. Nikon C2 Confocal Laser Microscope (Nikon, Tokyo, Japan) was used to collect the images with the merge channels of Cy3 and DAPI.

### Statistical analysis

All statistical analyses were performed using SPSS Statistics 13.0 (Chicago, IL, USA). Data were presented as mean ± SEM of at least three independent experiments and statistical significance of the differences between groups was calculated using the Student’s *t* test. All tests were two-sided and *P* < 0.05 was considered as statistically significant.

## Results

### MyoD1 suppresses GC cell migration and invasion in vitro and in vivo

**Fig. 1:**
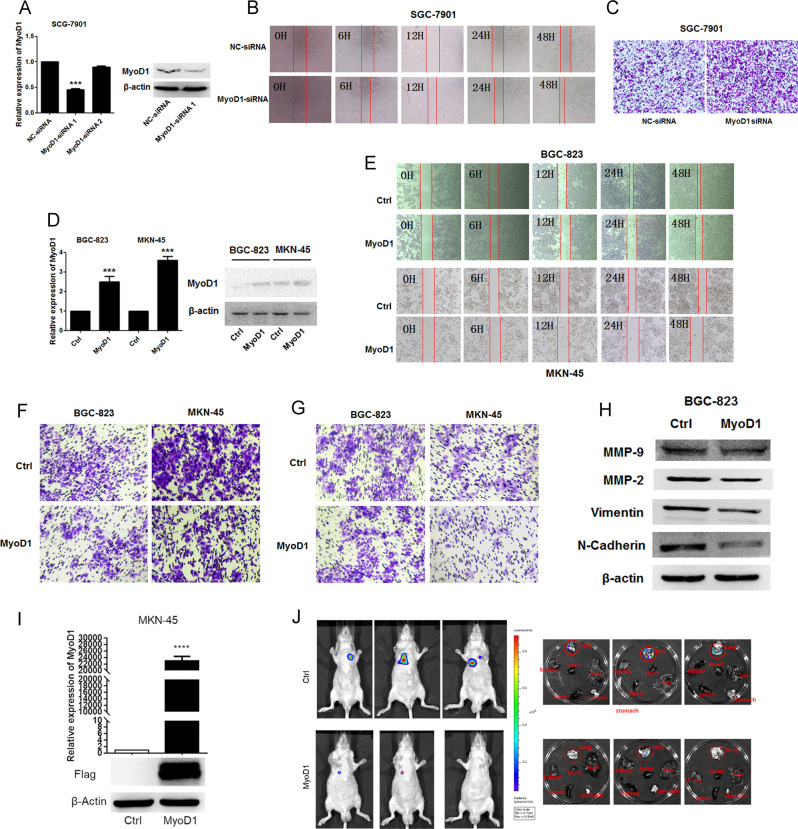
MyoD1 suppresses GC cell migration and invasion in vitro and in vivo. **a** qRT-PCR and western blotting analysis of MyoD1 in SGC-7901 cells transfected with NC-siRNA and MyoD1 siRNA. The effects of knockdown of MyoD1 on SGC-7901 cell migration were determined by wound-healing assay at 0, 6, 12, 24, and 48 h (**b**) and Transwell assay (**c**). **d** qRT-PCR and western blotting analysis of MyoD1 in BGC-823 and MKN-45 cells transfected with empty vector and MyoD1 overexpression vector. **e** The effects of MyoD1 overexpression on GC cell migration were determined by wound-healing assay at 0, 6, 12, 24, and 48 h. The effects of MyoD1 overexpression on GC cell migration (**f**) and invasion (**g**) were determined by Transwell assay without or with Matrigel. **h** Western blotting analys×is of the levels of MMP-2, MMP-9, vimentin, and N-cadherin in MyoD1-overexpressed GC cell lines compared with the controls. **i** qRT-PCR and western blotting analysis of MyoD1 in MKN-45 cells transfected with lentiviral vector LV-MyoD1-MKN-45 and control (LV-CN). **j** Small animal imaging and metastasis-bearing lungs and other organs imaging analysis to assess tumor metastasis at day 21 after injecting. The significant differences were analyzed according to t-test (***p < 0.001; ****p < 0.0001).

As we have shown, expression of MyoD1 was significantly lower in GC tissues compared with normal gastric tissues [11]. In this study, cell migration and invasion were measured using a wound-healing assay and transwell assays with SGC-7901 cells transfected by NC-siRNA or MyoD1 siRNA (Fig. 1a), and BGC-823 and MKN-45 cells transfected by null plasmid or MyoD1-containing plasmid (Fig. 1d). Down-regulation of MyoD1 expression promoted cell migration and invasion (Fig. 1b, c), whereas overexpression of MyoD1 suppressed cell migration and invasion (Fig. 1e–g). The levels of MMP-2, MMP-9, vimentin, and N-cadherin were decreased in MyoD1-overexpressed GC cell vs. control (Fig. 1h). To examine the role of MyoD1 in GC progression in vivo, we inoculated LV-MyoD1-MKN-45 and control cells into nude mice, respectively, and examined tumor distribution and size on the 21st day after injection. qRT-PCR and western blotting showed that both mRNA and protein of MyoD1 were significantly upregulated in LV-MyoD1-MKN-45 cells compared to the control cells (Fig. 1i). Both the distribution and the size of tumor were remarkably reduced by MyoD1 overexpression (Fig. 1j).

### MyoD1-binding DNA fragments are detected using ChIP-Seq

**Fig. 2:**
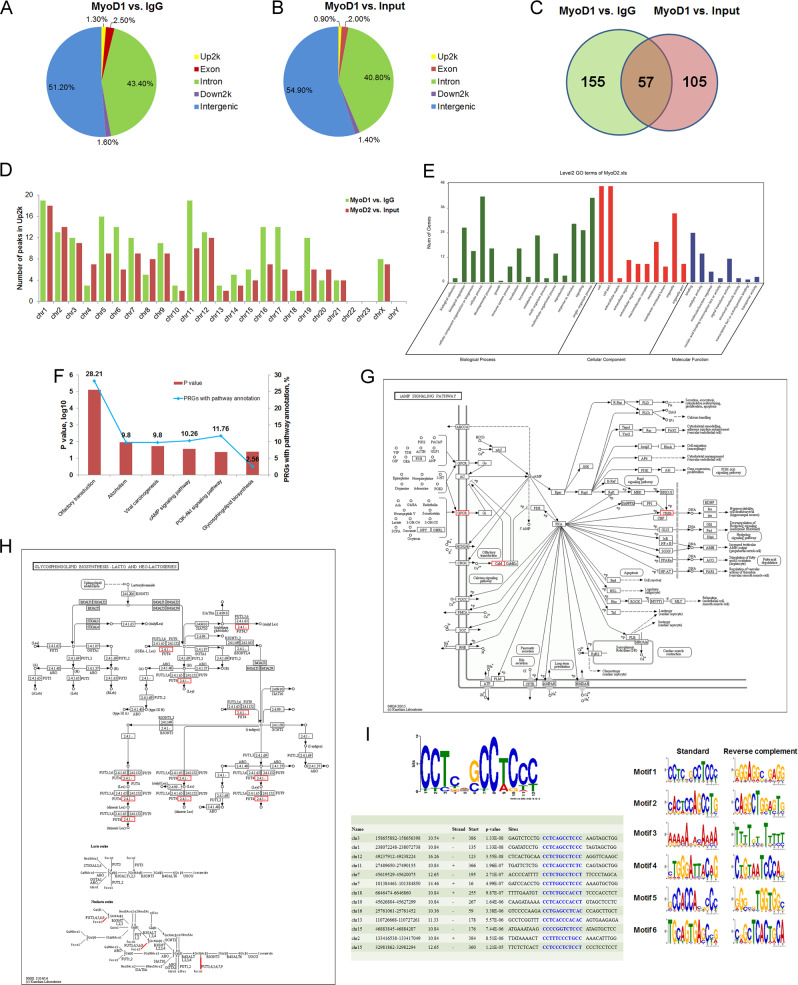
MyoD1-binding DNA fragments detected by ChIP-sequencing. **a**, **b** The distribution of peaks in the Up2k, Down2k, intergenic, intron, and exon regions analyzed according to MyoD1 vs. IgG (M1) and MyoD1 vs. Input (M2). **c** The number of peaks in the Up2k region that exhibited >2-fold enrichment over M1 and M2 and in common. **d** The number of peaks in the Up2k region on different chromosomes in M1 and M2. **e** GO annotation of 57 genes possibly bound by MyoD1 in their Up2k regions in terms of biological process, cellular component, and molecular function. **f** *P* value and peak-related genes (PRGs) with pathway annotation displayed in six enriched KEGG pathways (*p* value < 0.05). cAMP signaling pathway (**g**) and glycosphingolipid biosynthesis—lacto and neolacto series pathway (**h**) and their PRGs. **i** The specific nonredundant consensus sequences with *E* value < 10 and position *p* value < 0.0001.

We performed ChIP-Seq assays to identify target genes of MyoD1 in MKN-45 cells. Only about 1% DNA sequence was found in the region of 2 kb upstream of the transcription start site (Up2k) of genes bound by MyoD1 (Fig. 2a, b). A total of 212 and 162 ChIP-Seq peaks exhibited over 2-fold enrichment in MyoD1 vs. IgG (M1) and MyoD1 vs. input (M2) in Up2k, respectively. Among these peaks, 57 were common to M1 and M2 (Fig. 2c and Table S1). To characterize the chromosome distribution of ChIP peaks, we counted the number of peaks in Up2k with over 2-fold enrichment present on each chromosome. MyoD1 exhibited the highest binding strength to chromosomes 1, 5, and 11, and there were no peaks for chromosomes 22, 23, and Y (Fig. 2d). Gene ontology (GO) annotation showed that most of 57 gene products participate in cellular process and are located in cells with binding ability (Fig. 2e). These genes were also mapped to six KEGG (Kyoto Encyclopedia of Genes and Genomes) pathways with *p* value < 0.05 compared with the background signal of the human genome. The identified KEGG pathways included viral carcinogenesis, cAMP signaling pathway, glycosphingolipid biosynthesis—lacto and neolacto series, and others (Fig. 2f–h). Furthermore, among 57 DNA sequences, our data revealed a specific nonredundant consensus sequence CCTC[AT][GC]CC[TA]CC[CT] that appeared frequently in 13 of the 57 sequences with an *E* value < 10 and the position *p* value < 0.0001 (Fig. 2i). The six specific nonredundant consensus sequences and their reverse complementary sequences are listed in Fig. 2i.

### MyoD1 inhibits FUT4 transcription by binding its promoter

**Fig. 3:**
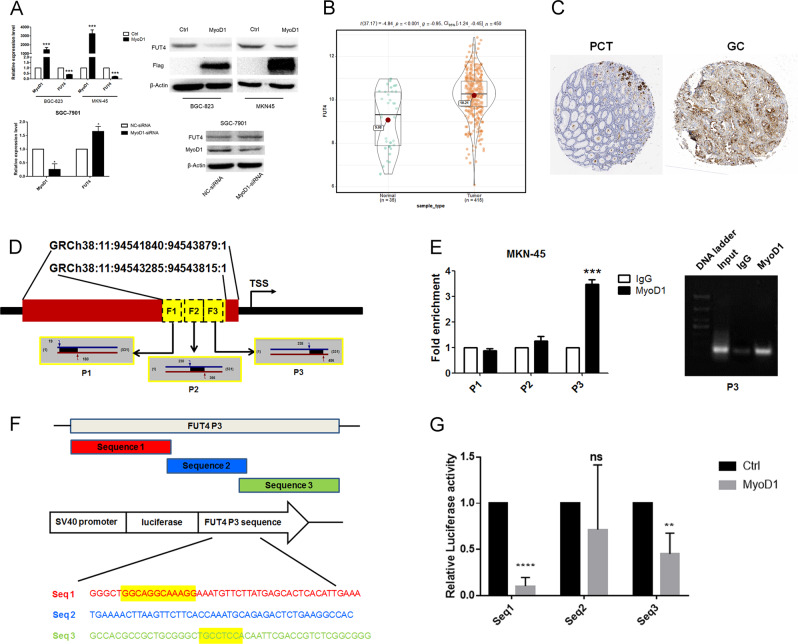
MyoD1 inhibits FUT4 transcription by binding its promoter. **a** Relative expression levels of MyoD1 and FUT4 in LV-MyoD1-BGC-823 and LV-MyoD1-MKN-45 cells vs. control cells and in SGC-7901 cells in which MyoD1 was silenced MyoD1 expression was measured by qRT-PCR and western blotting. **b** Significant high expression of FUT4 mRNA in 415 GC vs. 35 normal gastric tissues according to the TCGA database (*p* < 0.001). **c** Higher expression of FUT4 in GC than in normal gastric tissues detected by IHC and according to the Human Protein Atlas. **d** Prediction of the promoter region of *FUT4* and design of three pairs of primers (P1–P3) targeting fragments 1–3 (F1–F3) that covered full-length MBS of *FUT4*. **e** ChIP-qPCR of P1–P3 performed with anti-MyoD1 antibody. **f** Design of luciferase reporter for further validation of the direct binding by MyoD1 on MBS of *FUT4* gene. Specific motifs in sequences are marked by yellow rectangles. **g** Luciferase activities for sequence 1, 2, and 3 were compared between pCMV2-GV146-*MYOD1*-transfected cells and pCMV2-GV146-transfected cells. The significant differences were analyzed according to t-test (*p < 0.05, **p < 0.01, ***p  < 0.001, ****p < 0.0001, *n.s*. not significant).

On the basis of ChIP-Seq results, the DNA sequence GRCh38:11:94543285:94543815:1 in the Up2k region of *FUT4* with 7.3-fold enrichment was probably a MBS. To reveal a correlation between MyoD1 and FUT4, we measured their relative expression levels in LV-MyoD1-BGC-823, LV-MyoD1-MKN-45, and control cells. Overexpression of MyoD1 induced significant decreases in FUT4 mRNA and protein, whereas knockdown of MyoD1 produced the opposite effects (Fig. 3a). According to the TCGA database and the Human Protein Atlas [23], FUT4 was highly expressed in GC tissues compared with normal gastric tissues (Fig. 3b, c). The promoter region on *FUT4* was predicted using ECR Browser [24]. A highly conserved sequence (similarity is 93.1% with rheMac2) about 2040 bp upstream of the transcription start site (GRCh38:11:94541840:94543879:1) was considered as the promoter region of human *FUT4*, containing the identified potential MBS by ChIP-Seq (Fig. 3d). To validate the targeted relationship of MyoD1 and FUT4, we performed quantitative (ChIP-qPCR) with three pairs of primers (P1–P3) (Table 1) targeting fragments 1–3 (F1–3) that covered full-length MBS of *FUT4* (Fig. 3d). P3 showed a 3- to 4-fold enrichment in MyoD1 signal over non-specific IgG signal in MKN-45 cells (Fig. 3e). To further validate the direct MBS on *FUT4* gene, we used a luciferase reporter in HEK293 cells. We divided F3 into three sequences (sequences (Seq) 1–3) that overlapped adjacent sequences and cloned the sequences downstream of the luciferase reporter (Fig. 3f). We also compared Seq 1–3 and their complementary sequences to the specific nonredundant consensus sequences. Coincidentally, the motif 5′-GGCAGGCAAAGG-3′ in Seq 1 (yellow rectangle) was exactly matched with the complementary sequence of the most specific consensus sequence CCTC[AT][GC]CC[TA]CC[CT] (motif 1), that is, [GA][GA][GC][AT]GG[CG]xxAGG (x can be any base) (Figs. 2i and 3f). Motif of GCCTCCAin Seq 3 (yellow rectangle) was partially matched with the motif 5 (Figs. 2i and 3f). There was no motif in Seq 2 that could be matched to any specific nonredundant consensus sequence (Fig. 3f). Luciferase activities for Seq 1 and 3 were significantly decreased in pCMV2-GV146-*MYOD1*-transfected cells vs. pCMV2-GV146- transfected cells, whereas there was no change in luciferase activity with Seq 2 (Fig. 3g). These results were in accordance with the results of the specific nonredundant consensus sequence with these sequences, demonstrating that MyoD1 inhibited FUT4 transcription by directly binding to F3, especially to F3 Seq 1, in the *FUT4* promoter.

### MyoD1 overexpression alters UEA-I binding glycopattern in GC cells

**Fig. 4:**
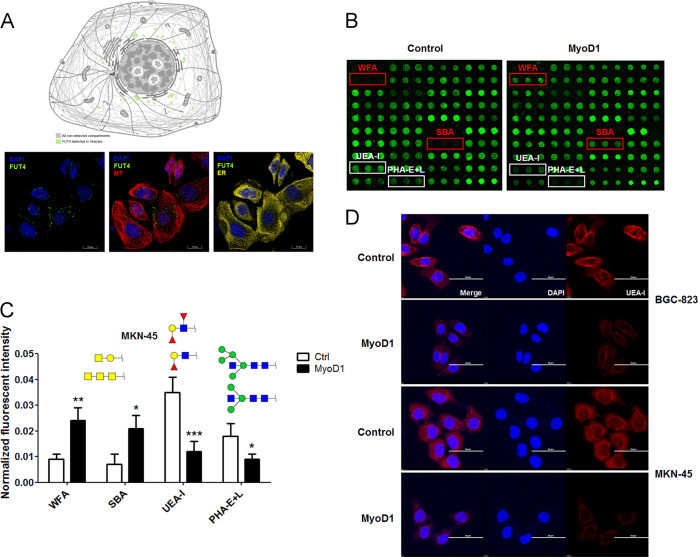
MyoD1 overexpression alters UEA-I glycopattern binding in GC cells. **a** FUT4 was mainly expressed in intracellular vesicles according to the Human Protein Atlas. **b** The glycopatterns of total glycoproteins from control and LV-MyoD1-MKN-45 cells defined by lectin microarrays. Fluorescent images were scanned at 70% photomultiplier tube and 100% laser power settings with a Genepix 4000B confocal scanner. A portion of the slide with three replicates of the lectin array is shown. The microarrays revealed increased normalized fluorescent intensities (NFIs, marked with red frames) and decreased NFIs (white frames) when LV-MyoD1-MKN-45 was compared with control MKN-45 cells. **c** NFIs and recognized glycans of lectins showed increased and decreased signals in LV-MyoD1-MKN-45 vs. control MKN-45 cells from lectin microarray (ratio >1.5 or < 0.67); the significant differences were analyzed according to *t* test (*p < 0.05, **p < 0.01, ***p < 0.001). **d** Lectin immunofluorescence approach to validate and investigate lectin binding sites in controls and LV-MyoD1-BGC-823 and LV-MyoD1-MKN-45 cells. The images were acquired with Cy3, DAPI, and merge channels (×120 objective magnification).

FUT4 was expressed in intracellular vesicles according to the Human Protein Atlas [23] (Fig. 4a). To examine whether the glycopattern of GC cells was altered when FUT4 was transcriptionally repressed by MyoD1, we extracted total native protein from control and MyoD1-overexpressed MKN-45 cells and probed the extracts with lectin microarrays (Fig. 4b). The normalized fluorescent intensities (NFIs) and the sugar-binding specificities for each of 37 lectins are summarized in Table S2. The differentially expressed glycans recognized by four lectins are shown in Fig. 4c. Terminal GalNAc, GalNAcα/β1−3/6Gal and (GalNAc)n recognized by WFA (*Wisteria floribunda*) and SBA (soybean) were significantly increased (fold change ≥ 2.0, *p* < 0.05). Conversely, we observed significant decreases in Fucα1-2Galβ1-4GlcNAc and Lewis antigens recognized by UEA-I and bisecting GlcNAc, bi- and tri-antennary complex-type N-glycan with outer Gal recognized by PHA-E + L (fold change ≤ 0.5, *p* < 0.05) in MyoD1-overexpressed vs. control MKN-45 cells (Fig. 4c). To further validate the differential expression and distribution of Lewis antigens recognized by UEA-I, we performed the lectin fluorescent staining with MyoD1-overexpressed and control GC cells. UEA-I showed strong binding to the plasma membranes of both GC cell types, whereas the binding decreased in the same region of MyoD1-overexpressed GC cells (Fig. 4d), suggesting that MyoD1 inhibits Lewis antigens expression by suppressing FUT4 transcription.

### Knockdown of FUT4 suppresses GC cell migration and invasion

**Fig. 5:**
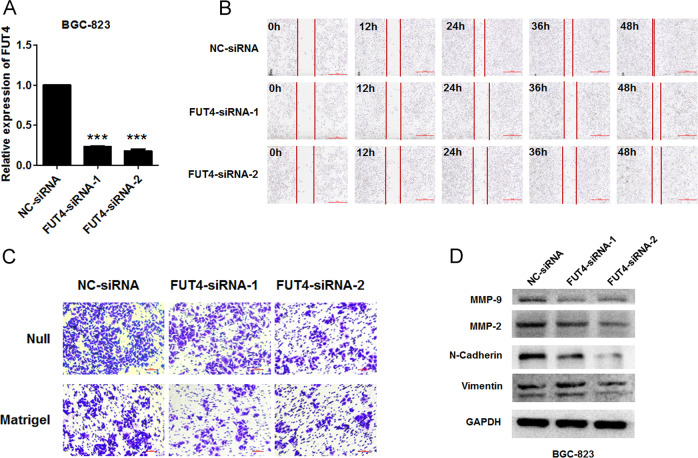
Knockdown of FUT4 suppresses GC Cell migration and invasion. **a** qRT-PCR analysis of FUT4 in BGC-823 cells transfected with NC-siRNA, FUT4 siRNA-1, and FUT4 siRNA-2. **b** The effects of knockdown of FUT4 on BGC-823 cell migration were determined by wound-healing assay at 0, 12, 24, 36, and 48 h. **c** The effects of knockdown of FUT4 on BGC-823 cell migration (up) and invasion (down) were determined by Transwell assay without (null) or with Matrigel. **d** Western blotting analysis of the levels of MMP-2, MMP-9, vimentin, and N-cadherin in knockdown of FUT4 on BGC-823 cells compared to the control. The significant differences were analyzed according to t-test (***p < 0.001).

To determine the function of FUT4, we synthesized FUT4-targeting siRNAs; FUT4 siRNA-1 and siRNA-2 efficiently knocked down FUT4 mRNA (Fig. 5a). Both wound-healing and transwell assays demonstrated that silencing FUT4 suppressed cell migration and invasion of BGC-823 cells (Fig. 5b, c). In addition, the levels of MMP-2, MMP-9, vimentin, and N-cadherin decreased in FUT4-silenced BGC-823 cells (Fig. 5d). The effects of FUT4 knockdown on cell migration and invasion and the molecular expression were in accordance with that of MyoD1 overexpression in GC cells, which implied that MyoD1 suppressed cell migration and invasion by inhibiting FUT4/MMPs signaling pathway.

## Discussion

MyoD1 is widely known to cooperatively establish the skeletal muscle phenotype by regulation of proliferation, promoting irreversible cell cycle arrest of precursor cells, and activation of sarcomeric- and muscle-specific genes to facilitate differentiation and sarcomere assembly [1]. Little is known about a relationship between MyoD1 and gastrointestinal cancers. Both our previous study and this study showed that expression of MyoD1 was significantly lower in GC cells compared with normal gastric tissues and cells. This low expression of MyoD1 induced apoptosis and suppressed migration and invasion in GC cell lines [11]. Because MyoD1 is a helix–loop–helix DNA-binding protein, we used ChIP-Seq to analyze the target DNA sequences that were bound by MyoD1. We identified 57 sequences in Up2k regions of genes involved in viral carcinogenesis, cAMP signaling pathway, glycosphingolipid biosynthesis—lacto and neolacto series, and other signaling pathways. We did not determine whether these genes are suppressed or activated by MyoD1.

Glycan-based interactions are important in immune surveillance, cell–cell adhesion, and cell–matrix interaction, contributing to treatment failure in tumor. The glycosylation form and density of glycans on a protein can be altered significantly by changes in cellular pathways and disease processes such as malignancy. FUT4 catalyzes the formation of type II Lewis antigens and is highly expressed in GC tissues and serum compared with chronic gastritis and gastric ulcer [25]. In GC cells, FUT4 is highly expressed on gastric cell surfaces and is regulated by transcription factors HSF1 and SP1 [17]. Our study revealed that FUT4 transcription was inhibited by MyoD1 binding to F3 of GRCh38:11:94543285:94543815:1 in the Up2k region of *FUT4*. Other studies have revealed that FUT4 is a novel regulator of EMT and is closely related to metastases and progression of colorectal cancer, hepatocellular carcinoma, and non-small-cell lung cancer [14, 26, 27]. We found that knockdown of FUT4 suppressed GC cell migration and invasion and decreased the levels of MMP-2, MMP-9, vimentin, and N-cadherin. These findings coincided with the effects of MyoD1 overexpression in GC cells and implied that MyoD1 suppressed cell migration and invasion by inhibiting FUT4/MMP signaling pathway.

Knockdown of FUT4 decreased expression of type 2H, LeX, LeY, and sialyl-LeX antigens. UEA-I binds to the α-1-2-fucosylated lactodifucotetraose-derived neoglycolipid or glycoproteins that contain type 2H and Le(y) [28]. Both lectin microarray and lectin immunofluorescence showed weakened binding of UEA-I to the plasma membrane of MyoD1-overexpressed GC cells, which was probably due to the inhibition of FUT4 by MyoD1. LeX antigen, also known as CD15 or stage-specific embryonic antigen-1, is a well-characterized cell surface trisaccharide with the structure Galβ1-4[Fucα1-3]GlcNAc, involved in many recognition processes [29]. LeX is synthesized by glycosyltransferases in the Golgi compartment, with the final step of transfer of l-fucose to *N*-acetylglucosamine by FUT4 or FUT9, depending on cell type [30]. LeX was overexpressed in colorectal cancer and leukemia, and the high levels of LeX were associated with chemo-resistance and poor prognosis [29]. By increasing glycosylation, FUT4 can activate α5β1-mediated sequential signal transduction and accelerate adhesion and invasion between integrin α5β1 in leukemia cells and fibronectin in the extracellular matrix [15]. Overexpression of CD15 or CD15s epitopes led to increased adhesion of cancer cells to cerebral endothelial cells compared with wild-type and cells with silenced CD15 or CD15s (*p* < 0.01); this increased adhesion could potentiate the transmigration of circulating NSCLC cells into the brain [27]. Overall, both FUT4 and its product, type II Lewis antigens, promote migration and invasion by various cancer cells, especially GC cells.

In conclusion, we demonstrated that, by directly binding to the F3 region in the Up2k of *FUT4* gene, MyoD1 suppresses migration and invasion of GC cells by inhibiting FUT4/type II Lewis antigens. This finding reveals downstream target genes of MyoD1 and suggests a novel regulatory relationship between MyoD1 and FUT4 in GC cells.

## Supplementary information

ChIP-Seq peaks exhibited over 2 folds enrichment in MyoD1 vs. igg (M1) and MyoD1 vs. input (M2) and the common.

The normalized fluorescent intensities (NFIs) and the sugar-binding specificities for each of the 37 lectins from two groups (control and MyoD1) detected using lectin microarray.
